# Understanding Factors Influencing Diabetic Patients’ Intention to Use Digital Health Services in Saudi Arabia Using the Technology Acceptance Model

**DOI:** 10.3390/ijerph21070889

**Published:** 2024-07-09

**Authors:** Tagreed Sadeek Al-Sulimani, Waad Bouaguel

**Affiliations:** 1 College of Business, University of Jeddah, Jeddah 23445, Saudi Arabia; tsalsilimani@uj.edu.sa; 2 LARODEC, ISG, University of Tunis, Le Bardo 2000, Tunisia

**Keywords:** technology acceptance model, diabetic, digital health

## Abstract

This study employs the Technology Acceptance Model to investigate the factors influencing Saudi Arabian diabetic patients’ intention to use digital health services. There is an urgent need to investigate the possibilities of digital health services in managing diabetes given the startlingly rapidly increasing prevalence rate of diabetes in KSA. The study examines the variables affecting patients’ acceptance and desire to use digital health tools to manage their diabetes. The study employs the Technology Acceptance Model to ascertain the crucial factors that impact patients’ opinions regarding the usefulness and ease of use of digital healthcare technologies. The proposed model extends the traditional Technology Acceptance Model by adding two new constructs, perceived privacy and trust. These constructs were examined by analyzing the intentions of 600 respondents through online surveys. The study’s conclusions showed that attitudes toward using digital health services for KSA diabetic patients are greatly influenced by every component of the extended Technology Acceptance Model. The study’s conclusions add to the body of knowledge already in existence and offer insightful information to decision-makers hoping to improve digital health services.

## 1. Introduction

Diabetes mellitus is a chronic disease with an alarmingly fast-rising prevalence rate in the Kingdom of Saudi Arabia (KSA) [1]. With more than 1.8 million diabetic patients, KSA has one of the greatest rates of diabetes prevalence in the world, and it is predicted that this population will double with an increase of 105.4% by 2035 [2]. Al-Nozha et al. [3] presented the results of an epidemiological health survey conducted between 1995 and 2000, focusing on Saudi citizens aged between 30 and 70. The survey presented a full picture of the prevalence rate of diabetes mellitus in KSA, and the highest prevalence percentages are found in the northern and eastern regions of KSA, at 27.9% and 26.4%, respectively. The actual number of patients with diabetes is causing the expense of diabetes care in the KSA to rise. Diabetes was estimated to have cost KSA 1.5 billion dollars in 2010. After 2020, it is anticipated that public healthcare spending in KSA will exceed 6.5 billion dollars due to the expected rise in the number of individuals with diabetes [4]. In contrary to these economic difficulties in healthcare related to diabetes disease, KSA is accelerating the digitization of its healthcare sector. One of the Gulf regions where mobile phones, the Internet, and social networking are most widely used is KSA [5]. According to Yaakoubi [6], KSA is spending 6.4 billion dollars on new and emerging digital technology ventures. With 24.2 million users, KSA is third in the world in smartphone usage, and nearly 75 percent of the populace is smartphone users. There were about 39.53 million internet consumers worldwide in 2021. The percentage of people who use the internet exceeds 95%. In terms of the country’s online penetration, this is a remarkable number. The predicted internet usage rate for 2025 is expected to rise to 97%. Although the healthcare industry has seen growth and popularity recently, this rapid digitization of society has not yet been mirrored in the areas of diabetes care, where such technologies can significantly enhance patient support and healthcare delivery services. KSA can benefit from improved medical services and well-being thanks to digital health. The sector provides growth opportunities for both current market participants and new competitors. Diabetes technology refers to any technology used by people with diabetes to manage their medical condition [2]. Numerous studies have shown how beneficial modern technologies are in improving patient satisfaction overall and controlling diabetes [3]. According to Alhowaish [4], digital health includes not just the delivery of healthcare services such as medical consultation, monitoring, diagnosis, and treatment services but also digitally based solutions such as telemedicine tools, electronic health records, and medical apps, chronic care management tools, clinical decision support tools, and e-prescribing software.

With various services and solutions, digital health is currently recognized as a cutting-edge, dependable, and effective method of delivering healthcare. The ability of the digital health market to grow depends on a variety of enablers and hurdles related to law, culture, the state of the economy, and technology. Linking technology with issues of human behavior and perception is required to get a better knowledge of how the public is utilizing digital health services to overcome these obstacles and challenges. Instead of examining the factors encouraging people to accept digital health, many studies have focused on the investigation of the national benefits of digital health and the technical and infrastructural hurdles in implementing it [7,8]. By identifying the variables influencing diabetic patients’ actual use and intention to use digital health services, this research contributes to the body of literature already available. Decision-makers who want to improve the digital health services offered in KSA can benefit from the knowledge it provides.

## 2. Theoretical Framework of the Technology Acceptance Model and its Use in the Literature for Healthcare

Following the COVID-19 pandemic spread, diabetes patients in KSA become more and more accepting of digital health tools. To address the effects of the global pandemic, it was decided that the utilization of remote internet consultations “E-consultations” needed to exponentially increase [9] the advantages of employing digital health technologies for managing diabetes, such as better access to healthcare, improved self-management of their illness, and more convenience, were becoming more and more clear to patients. In 2020, the KSA Ministry of Health performed an investigation in which they found that 75% of individuals with chronic illnesses, including diabetes, were interested in using telemedicine services. The study also discovered that 62% of patients were willing to give healthcare providers online access to their personal health information [8,9,10]. Overall, the availability of digital health technologies and understanding of their potential advantages have helped to increase their public acceptance among Saudi patients with diabetes. Creative solutions to providing health care through the use of technology may face some opposition, nevertheless, as a result of issues with systemic trust, ignorance of its advantages, digital illiteracy, and the complexity of usage [11].

The last ten years have seen substantial research into the factors that influence how healthcare technologies are used. Different models and theories of technology acceptability were used to study these factors. AlQudah et al. [12], examined the literature on technology adoption in healthcare. From a total production of 1768 studies, 142 empirical studies met the requirements and underwent in-depth analysis. The main conclusions indicated that The Technology Acceptance Model (TAM) and the Unified Theory of Acceptance and Use of Technology (UTAUT) are the most used models for analyzing factors influencing how different healthcare technologies are accepted by diverse user groups, situations, and nations. TAM is an important model for understanding and predicting users’ acceptance of new technology or information systems, including digital healthcare technologies. TAM is one of the most common worldwide models [13], it is based on the idea that users’ behavioral intentions of using technology are influenced by their opinions of how valuable and simple the technology is to use. TAM can serve as a helpful framework for analyzing the elements that affect patients’ intentions to adopt digital healthcare technology for managing diabetes in KSA. TAM can specifically assist in identifying the critical variables influencing patients’ views of the value and usability of digital healthcare technology, including mobile apps and telemedicine services.

## 3. Proposed Model and Hypotheses

### 3.1. Perceived Usefulness, Perceived Ease of Use and Attitude

The Theory of Reasoned Action in Social Psychology [14,15], which maintains that behavioral intention is a powerful predictor of actual action, served as the foundation for the traditional TAM. Numerous studies examining potential users’ behavioral intention to utilize a specific technology have employed the TAM as a conceptual framework. “The degree to which a person has formulated conscious plans to perform or not perform some specified future behavior” is the definition of behavioral intention [16]. Perceived utility (PU) and perceived ease of use (PEOU) are two design features or antecedents that impact user acceptance behavior in the traditional TAM, which focuses on employing technology. According to TAM, behavioral intention to embrace the new technology determines how the system is used. The user’s attitude toward using the system, as well as its PU and PEOU, all have an impact on the behavioral intention. The degree to which someone utilizes the system by valuations is referred to as their attitude toward utilization.

In technology acceptability and healthcare, much research has been done on the relationship between PU and the intention to use digital health services [17,18]. According to Hong et al. [19], elderly Koreans’ inclination to adopt IoT HealthCare services is significantly influenced by PU. The research conducted by Ha and Park [20] also indicates that seniors with many chronic diseases have good technology adoption and readiness to use it in healthcare.

According to Davis [21], PU is the extent to which an individual thinks utilizing a specific system would improve his or her job performance. The PU of digital health services in the context of diabetic patients refers to how these services are viewed to improve diabetes management, offer individualized support, improve access to healthcare providers, or provide practical self-monitoring tools. Zin et al. [18], confirmed that the PU positively impacts patients with chronic illnesses’ behavioral intentions toward accepting smartphone health technologies for managing their conditions.

The term “ease of use” describes how simple it is to use technology. People tend to view a system as more valuable if it is simple to use. An individual’s intention to use the system is also influenced by their perception of its utility and ease of use.

Davis [21] recognized PU and PEOU as the primary criteria influencing people to embrace and continue using new technology in the original version of TAM. He described PU and PEOU as a person’s confidence level in a technology’s ability to help them and be simple to use. The terms “PU” and “PEOU” relate to the expected benefits and level of effort associated with digital health wearables, respectively [22]. PEOU of digital health services in the context of diabetic patients refers to how these services are viewed as easy to understand, operate, and maintain [23]. The following hypotheses were created from the extension of TAM based on the previously indicated analysis.

**Hypothesis 1**  (H1). *The Perceived attitude positively influences the intent to use Digital health services for diabetic patients in KSA.*

**Hypothesis 2**  (H2). *PU positively influences the attitude toward using Digital health services for diabetic patients in KSA.*

**Hypothesis 3**  (H3). *PEOU positively influences the attitude toward using Digital health services for diabetic patients in KSA.*

### 3.2. Perceived Privacy and Perceived Trust

Because classical TAM frequently fails to capture many important elements unique to the technology’s setting, many researchers often expand TAM by incorporating external components [15]. According to Wu and Chen [24], privacy and trust are among the characteristics that have been introduced as significant factors influencing users’ acceptance of information technology and are thus necessary for assessing new systems. Furthermore, previous research indicates that users’ attitudes toward the technologies are positively influenced by perceived privacy and trust, which play crucial roles in their acceptance [25].

Many users of digital healthcare services worry about their data privacy, [26,27]. Concerns about losing one’s privacy and the necessity for safeguards against unauthorized disclosure and use of personal data are the two main definitions of privacy concerns [28]. It is also concerned with managing the flow of personal data, ensuring that individuals in possession of the data utilize it appropriately, respecting an individual’s desires, and keeping sensitive information private. It implies that unless the individual has granted permission or it is essential, professionals or apps shouldn’t provide personal information about them to third parties [27]. People are more concerned about privacy when information is used without their knowledge or agreement or when its intended use is unclear.

Moreover, privacy has been also defined as the right to stop personal information from being disclosed to third parties [15]. Subsequent research has shown that privacy is multifaceted and encompasses informational privacy in addition to accessibility, physical, and expressive privacy. “How, when, and to what extent information about the self will be released to another person” is known as informational privacy. The term “acquisition or attempted acquisition of information that involves gaining access to an individual” refers to accessibility and privacy. Physical privacy is “the extent to which an individual is physically accessible to others,” such as when someone views another person’s screen without authorization. “Protects an area for expressing one’s self-identity or personhood through speech or activity” is the definition of expressive privacy [15].

The body of research in healthcare that addresses the patient absence of privacy concerns has grown dramatically [29,30]. For instance, Hoque et al. [31] included the privacy component into TAM and discovered that this factor significantly influenced Bangladeshi citizens’ behavioral intentions to adopt e-Health. Furthermore, in the case of China’s hospitals, the absence of privacy was found to be a significant behavioral intention factor for using e-Health technologies [32]. The absence of privacy factors could harm Saudi Arabian diabetes patients’ attitudes toward using digital healthcare. The following theory was created from the extension of TAM based on prior studies.

**Hypothesis 4**  (H4). *Perceived privacy positively influences the attitude toward using Digital health services for diabetic patients in KSA.*

Users often undertake tasks that put them in danger in the hopes that the service provider on the other end will fully follow the set of protocols to complete a transaction. Trust plays a significant role in this. Since users in a virtual environment do not influence how their activities turn out, trust becomes essential for them to have a strong conviction in the other party’s dependability [33]. Studies have indicated that trust significantly and favorably affects attitudes and intentions to use systems [34]. Users who are more trusted are less likely to doubt the legitimacy of internet services.

Many types of research have shown that patients’ behavioral intention toward the e-Health system was positively influenced by trust [32,35]. “A person’s practical expectation that another party possesses the characteristics of trustworthiness, which in turn, guides decision making” is the definition of trust in the context of technology adoption.

**Hypothesis 5**  (H5). *Perceived trust positively influences the attitude toward using Digital health services for diabetic patients in KSA.*

**Figure 1:**
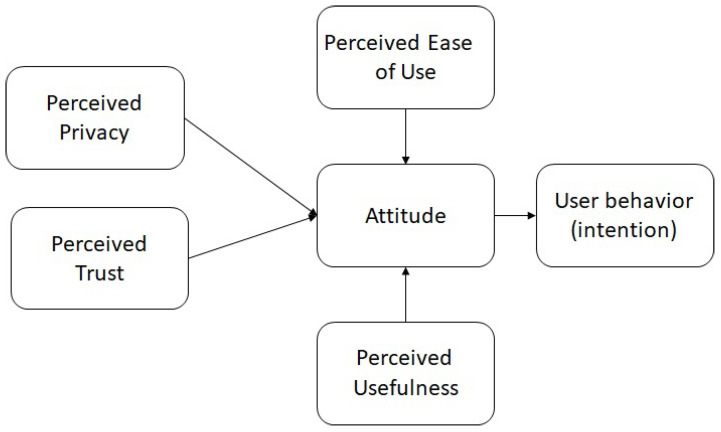
Theoretical framework.

Figure 1 presents the different hypotheses proposed in this study.

## 4. Materials and Methods

We used in this investigation convenience sampling. Studies in the fields of medicine and qualitative research frequently employ convenience sampling. Convenience sampling in medical research typically entails choosing clinical cases or volunteers around a specific site or a medical records database. The study’s data set was restricted to the Jeddah region. To determine the group of diabetes patients, we targeted those receiving treatment at the University of Jeddah healthcare facilities.

Our study’s objectives were addressed in a thorough and well-structured survey questionnaire. It includes pertinent questions about the patient’s experiences, opinions, and intentions regarding digital health services for managing diabetes. To maintain confidentiality and anonymity we avoided collecting any personally identifiable information. We then created and distributed the online surveys using Google Forms™, which allowed us to create and distribute surveys electronically. We tested the survey on various devices and browsers to ensure compatibility before distributing it, and we shared it using the contact information obtained from the University of Jeddah healthcare institutions to send the survey invitation electronically using the university’s online platforms.

Convenience sampling is frequently employed in qualitative research, particularly in the social sciences and education, where it is practical to use pre-existing groups, and it is commonly used to create hypotheses that can be investigated in more detail in future research or to gain a sense of people’s attitudes and beliefs, therefore we believe it could be a suitable fit for our study. 600 respondents completed the survey between 1 March 2023 and 1 January 2024

There were two parts to the survey. In the first part, questions about the participants’ socio-demographic information were asked. The study model’s variables were covered in the second part of the survey. Participants’ answers were recorded using five-point Likert-type scales (1 = Strongly Disagree, 2 = Disagree, 3 = Undecided, 4 = Agree, and 5 = Strongly Agree).

Table 1 presents socio-demographic characteristics.

Table 2 consists of several Likert scale items related to different aspects of the study. Each item is rated on a scale from one to five, representing different levels of agreement or disagreement. By analyzing the responses to these Likert scale items, researchers can gain insights into participants’ perceptions, attitudes, and intentions toward using a diabetes digital healthcare service. These findings will serve in evaluating the potential acceptance and adoption of such services in managing diabetes.

## 5. Data Analysis and Results

### 5.1. Data Analysis

Psychometrics and statistical analysis are usually carried out in a research study’s data analysis stage. Following data gathering, this step entails analyzing the gathered information to derive relevant findings and inferences.

Analyzing and interpreting data using quantitative data such as numerical measurements or survey responses in research studies typically involves statistical analysis. Descriptive statistics, such as mean and standard deviation, summarize the data.

Results in Table 3 provide the key statistics for the study constructs, including the mean, standard deviation, and Cronbach’s Alpha coefficient. The mean scores for each construct range from 3.5 to 3.8, indicating that the participants generally had a positive perception of the constructs being measured. The standard deviations range from 1.1 to 1.3, suggesting that there is some variability in the responses among participants. The larger standard deviations imply that there were some differences in the participants’ viewpoints or attitudes within each domain.

A more complete understanding of the participants’ responses can be obtained by considering both the mean scores and standard deviations collectively. The standard deviations show the degree of variety in those replies, but the positive mean scores show a generally positive perception.

Descriptive statistics provide an overview of a data set’s features, whereas inferential statistics assist you in determining whether your data supports or contradicts your hypothesis and whether it can be applied to a wider population. Regression analysis and hypothesis testing are the two main categories of inferential statistics. In this work, we focused on hypothesis testing, we analyzed the data using structural equation modeling (SEM), which has the advantage of being able to examine multiple dependent relationships at once, particularly in cases where the constructs in the model have both direct and indirect effects on one another. It is possible to measure latent variables using multiple indicators and test hypotheses at the construct level rather than the item; analyze relationships between latent and observed variables, and model random errors in the observed variables to provide more precise measurements. When using a two-step approach to SEM [36], the measurement model is measured in the first phase to determine how effectively the survey questions, or observed indicators, measure the latent (unobserved) components. The relationships between the exogenous and endogenous latent variables are measured in the second phase, which is the structural portion of the SEM. The models were analyzed with Smart PLS4.

In the first phase of evaluating the study hypothesis, we use psychometrics. This entails evaluating the instruments’ validity and reliability to make sure they accurately measure the target constructs. To evaluate and quantify psychological constructs like attitudes, PU, PEOU, etc., psychometrics is applied. Test results and assessments are analyzed using psychometrics to determine their validity, reliability, and interpretability. This makes it possible to guarantee that the assessment results have meaning and can be interpreted correctly.

Cronbach’s Alpha is a measure of internal consistency reliability, and it ranges from 0 to 1, with higher values indicating greater reliability. The Cronbach’s Alpha coefficients for all constructs are above the acceptable threshold of 0.7, ranging from 0.868 to 0.991, indicating that the measures used to assess each construct are reliable (see Table 3 and Table 4). The higher the alpha coefficient, the more the factors have shared covariance and measure the same underlying concept [23].

Overall, these results suggest that the measures used to assess the constructs are reliable and that the participants generally have positive perceptions of the constructs being measured.

Examining the alpha coefficient for each construct in detail reveals that all scores are in the range of 0.8 to 0.9, indicating great reliability. On the other hand, it’s feasible that our measurement items are becoming more and more redundant if we’re getting close to 1. Therefore, to rule out the redundancy hypothesis, we carefully examine Cronbach’s alpha if measurement items are eliminated.

Table 4 shows the reliability coefficients for each construct and the effect of dropping one item at a time from the construct. The reliability coefficients include Average Inter-item Correlation (average_*r*_), Cronbach’s Alpha (raw-alpha), and Standardized Cronbach’s Alpha (std.alpha). These coefficients reflect the extent to which the items in each construct are related to each other and measure the same underlying construct.

The obtained Cronbach’s alpha for each questionnaire question, if the item is eliminated, is presented in the fourth column of Table 4. This column provides useful information that can be used to identify the questionnaire items that add to the overall alpha and those that don’t. Our initial alpha for construct 1 (PU) was 0.93. Table 4 shows that the overall raw alpha will decrease from 0.93 to 0.9, 0.91, or 0.92 if any of the questions (P_*usefulness*1_, P_*usefulness*2_, P_*usefulness*3_, P_*usefulness*4_, P_*usefulness*5_) are removed. We may conclude that the latter questions are useful and add to the overall reliability of PU as the raw alpha for the PU construct will fall with their removal. The same conclusions were achieved for the remaining constructs.

### 5.2. Composite Reliability

According to Cohen [37], using only Cronbach’s alpha coefficient is insufficient to examine the scale construct validity. Therefore, it is more acceptable to investigate the internal consistency of the scale’s questions using a composite reliability score. Reliability refers to the consistency and stability of the measures used to assess a construct. Validity refers to the extent to which a construct measures what it is intended to measure. Convergent and discriminant validity tests can be used in this context.

To study convergent validity, Fornell and Larcker [38] suggested three methods to examine convergent validity. These include item reliability measured by Cronbach’s Alpha coefficient, composite reliability (CR), and Average Variance Extracted (AVE).

In Table 5, four measures of reliability and validity were reported: Cronbach’s Alpha, rho_*A*_, AVE, and CR. Buabeng [36] defines AVE as the grand mean value of the squared loadings of the indicators associated with the construct. AVE measures the amount of variance in the construct that is accounted for by its indicators, with values of 0.5 or higher being considered acceptable [38]. Rho_*A*_ is a measure of construct reliability that considers the correlations between the construct and other constructs in the measurement model. Generally, rho_*A*_ values of 0.7 or higher are considered acceptable. Based on the values in Table 5, it appears that all six constructs have good validity, as all of the AVE values are above 0.77 and all of the rho_*A*_ values are above 0.88. Nunnally and Bernstein [39] suggested values between 0.7 and 0.9 for CR to be considered satisfactory. As shown in Table 5, the CR of an item ranged from 0.930 to 0.979.

Overall, based on the values in Table 5, it appears that all six constructs have excellent reliability, as all the coefficients exceed 0.85, meaning that the measures used to assess these constructs have strong reliability and validity, which suggests that they are likely to be robust and accurate indicators for measuring Diabetic Patients’ Intention to Use Digital Health Services in KSA.

### 5.3. Discriminant Validity

According to Buabeng [36], the degree to which a construct differs from other constructs according to empirical standards is known as discriminant validity. Two procedures were proposed to evaluate discriminant validity: the loadings of each indicator should be greater than all of its cross-loadings, and the Fornell–Larcker criterion, which states that the square root of AVE of each latent construct should be greater than the highest squared correlations between any other construct [38].

The findings of the discriminant validity study are shown in Table 6. It displays the correlation coefficients between the various study variables or constructs. The diagonal elements represent the AVE for every construct. When the square root of the AVE for each construct is greater than the correlation coefficients between that construct and other components, discriminant validity is attained. The table’s information suggests that discriminant validity is attained since each construct’s square root of the AVE is greater than the correlation coefficients between that construct and other constructs. This implies that the study’s constructs are unique and measure various underlying ideas.

### 5.4. Hypotheses Testing and Path Analysis

**Figure 2:**
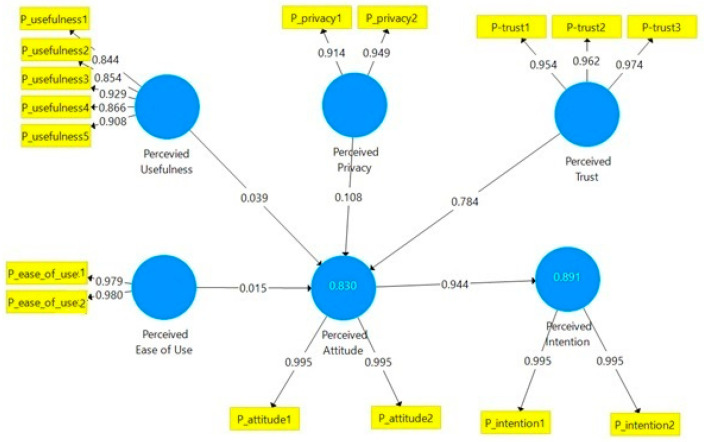
SmartPLS output of the structural model assessment.

We use Smart PLS software to create a structural model to fully investigate the underlying connections between constructs (see Figure 2). There are two endogenous variables and four external variables in our structural model. The values of the route coefficients β and the coefficient of determination $R^{2}$ are shown in Figure 2. The value in the dependent variables of the structural model represents the coefficient. This coefficient shows how much each exogenous component combined impacts the dependent variable. For example, PEOU, PU, Perceived privacy, and Perceived trust account for 83% of the difference in the Perceived attitude of diabetic patients in KSA regarding embracing digital health services. In KSA, the attitude construct accounts for 89.1% of the variation in patients’ Perceived intention to use digital health services.

The path coefficients are basically like linear regression weights. Path coefficients are usually used in the structural model to study the causal link between the exogenous and endogenous variables. The path coefficients generated by the structural model are also presented in Figure 2. The results imply a significant positive effect of all the factors. By having a positive relation between variables, this implies that increasing the value of the dependent variables will increase the value of the dependent one, meaning that

- If we increase one unit of Perceived trust, then 0.784 units of Perceived attitude will increase
- If we increase one unit of PU, then 0.039 units of Perceived attitude will increase
- If we increase one unit of PEU, then 0.015 units of Perceived attitude will increase
- If we increase one unit of Perceived privacy, then 0.108 units of Perceived attitude will increase
- If we increase one unit of Perceived attitude, then 0.944 units of Perceived intention increase

Hence, all the hypotheses are accepted.

## 6. Discussion

The purpose of this study was to investigate the potential of TAM integration, and to identify the variables affecting diabetes patients’ willingness to adopt digital health services in KSA. It was discovered that the perceived attitude had the biggest significant impact on the perceived intention based on the model shown in Figure 2. Perceived privacy, perceived trust, PU, and PEOU all accounted for 83% of the observed variations in the attitude of Saudi patients. This shows that the final model could describe the variables affecting diabetic patients’ willingness to use digital health services in KSA. The most significant factor influencing diabetic patients’ attitudes about using digital health services in KSA is perceived trust. Out of all the path coefficients to the attitude in the model, the one from perceived trust to attitude had the greatest value. This emphasizes how crucial it is for consumers to build positive trust around the use of technology.

For diabetic patients in KSA, establishing confidence in digital health care is a continuous process involving multiple critical tactics. To build trust, it is important to first communicate openly and honestly with patients about the gathering, storing, and use of personal health data. Health institutions and the government should also explain the rationale behind and advantages of adopting digital health services. They, also have to answer any worries or inquiries that patients may have. Second, when implementing those digital health systems, they should be intuitive, user-friendly, and simple to navigate. Think about the unique requirements and inclinations of Saudi diabetic patients. Patients’ comfort and trust in using the technology can be increased by a well-thought-out and user-friendly interface. Third, the involvement of medical professionals in the digital healthcare process, such as physicians or diabetes educators. Patients’ trust may be bolstered when they observe that their medical professionals encourage and promote digital health services. To reaffirm the importance of digital technologies, encouraging healthcare practitioners to actively recommend and interact with patients about digital tools is very important. Last, and not least just as importantly, give patients access to thorough instructional resources and support materials. Provide detailed instructions on the safe and effective use of digital health instruments. Provide patients with the information and tools they need to make wise decisions about their health. This educational support demonstrates a dedication to the well-being of the patient, which promotes trust.

Through the construct attitude, PEOU had a negligible indirect impact on intention. to develop PEOU in KSA’s digital health care system for people with diabetes. The government ought to Employ a user-centered design methodology in the process of creating digital health solutions. The created solution has an intuitive, aesthetically pleasing user interface. When users connect with the digital healthcare solution, developers must also give them clear instructions and direction. Provide users with interactive guides, or step-by-step tutorials to help them learn how to utilize the platform and complete tasks.

There are several perspectives and areas for future work in this field. Firstly, further research can explore the design features and functionalities of digital health technologies that diabetic patients in KSA most value. Understanding patients’ preferences and needs can inform the development of tailored and user-friendly digital health solutions. Additionally, future studies can investigate the impact of digital health services on patients’ health outcomes, such as glycemic control and quality of life. Longitudinal studies can provide valuable insights into the effectiveness and sustainability of digital health interventions. Moreover, exploring healthcare providers’ roles and attitudes toward digital health services can provide a comprehensive understanding of the healthcare ecosystem and potential strategies for successful implementation. Finally, considering the cultural and contextual factors specific to KSA can further enhance the knowledge of patients’ acceptance and utilization of digital health technologies in the country.

## 7. Conclusions

This research provides insights into the factors influencing the intention of diabetic patients in KSA to utilize digital health services. The study emphasizes the potential advantages of digital health technologies in enhancing healthcare accessibility, diabetes self-management, and patient convenience. To examine the effects of PEOU, PU, perceived trust, and perceived privacy on patients’ attitudes and intentions to use digital health, an extension of TAM was proposed. The findings highlight the significance of PU and PEOU as key factors influencing patients’ acceptance and intention to adopt digital health technologies. The study underscores the importance of increasing patients’ awareness and knowledge regarding the benefits of digital health services while addressing concerns related to trust and privacy. Overall, the study successfully achieved its six objectives, including exploring the factors influencing diabetic patients’ intention to use digital health services, investigating acceptance factors, extending TAM, assessing patients’ perceptions, contributing to existing knowledge, and providing valuable information for decision-makers to enhance digital health services for diabetic patients in KSA.

This study is significant and could benefit global public services and the scientific community. Firstly, by carrying out a study that is exclusive to KSA, researchers can concentrate on the distinct elements and difficulties that affect diabetes patients’ intentions to use digital health services in that particular setting. This approach makes it possible to comprehend the cultural, socioeconomic, and healthcare system-related factors that could have an impact on KSA’s acceptance of digital health services on a deeper level. Despite the study’s Saudi Arabian focus, its conclusions and new perspectives can help the scientific community to better comprehend the variables affecting diabetes patients’ acceptance of digital health services.

Second, TAM is a widely recognized paradigm for researching technology adoption that can be utilized in various international healthcare settings. As a result, the study’s findings may have implications and be applicable outside of KSA, providing academics and practitioners with global knowledge. Third, public services and policymakers can benefit from knowing the variables that affect diabetic patients’ inclination to use digital health services. Policymakers can create focused interventions and policies to encourage the use of digital health services by using the data to identify obstacles and enablers to their adoption. This has the potential to improve patient outcomes, healthcare delivery, and experiences—not only in KSA but in other countries dealing with comparable issues.

In summary, carrying out research relevant to KSA, can enhance the body of knowledge within the scientific community by supplying context-specific insights. It can also potentially assist public services globally by guiding policy changes and enabling comparison analysis.

## Figures and Tables

**Table 1:** Socio-demographic characteristics.

Attributes	Modalities	Frequency
Gender	Male	83.3%
	Female	16.7%
Nationality	Saudi	90%
	Non Saudi	10%
Age	18–24	6.7%
	25–34	26.7%
	35–49	40%
	50–64	20%
	65 and Above	6.7%
Occupation	Governmental Sector	60%
	Self-Employed	6.7%
	Private Sector Employee	23.3%
	Unemployed	10%
Education Level	Intermediate (Secondary, Post-Secondary)	56.7%
	Advanced (Master, Doctoral)	20%
	Level Not Stated	23.3%
Month Income(in Saudi Riyal)	1000–10,000	276
	Less than 5000	6.7%
	5001–10,000	10%
	10,001–15,000	10%
	15,001–20,000	33.3%
	More than 20,000	40%
Other Chronic Diseases	Yes	33.3%
	No	66.7%
History of Using Digital Health Service	Yes	56.7%
	No	43.3%

**Table 2:** Factors description for the study model’s variables.

Variables	Factors	Factors Definition
Perceived Usefulness	P_*Usefulness*1_	Using a diabetes digital healthcare service would increase the quality of my life.
	P_*Usefulness*2_	Using a diabetes digital healthcare service would be useful for me.
	P_*Usefulness*3_	Using a diabetes digital healthcare service would be beneficial for me.
	P_*Usefulness*4_	Using a diabetes digital healthcare service would be convenient for me.
	P_*Usefulness*5_	I would consider diabetes digital healthcare services as a useful means for controlling and monitoring my diabetes.
Perceived Ease of use	P_*easeofuse*1_	I believe a diabetes digital healthcare service would be easy for me to use.
	P_*easeofuse*2_	I believe the use of a diabetes digital healthcare service would be clear and understandable for me.
Perceived Privacy	P_*privacy*1_	I believe that the personal information I provide will be kept confidential.
	P_*privacy*2_	I am convinced that my personal information is shared privately and is stored in a way that unauthorized parties will not be privy to it
Perceived Trust	P_*trust*1_	I believe that digital diabetes applications and service results can be trusted.
	P_*trust*2_	Would you say that most digital services for diabetes people can be trusted.
	P_*trust*3_	Nowadays, you can rely on digital healthcare services for diabetes.
Perceived Attitude	P_*attitude*1_	I like the idea of using digital healthcare services to manage my diabetes.
	P_*attitude*2_	I have the desire to use digital healthcare services to manage my diabetes.
Perceived Intention	P_*intention*1_	If I had a digital healthcare service available, I would favor using it rather than conventional services.
	P_*intention*2_	I will recommend others to use digital healthcare services to manage their diabetes.

**Table 3:** Key statistics for the study constructs.

Attributes	Factors	Mean (M)	Standard Deviation (SD)	Cronbach’s Alpha
Perceived usefulness	P_*usefulness*1_	3.8	1.2	0.93
(M = 3:7; SD =1.1)	P_*usefulness*2_	3.8	1.2	
	P_*usefulness*3_	3.6	1.3	
	P_*usefulness*4_	3.6	1.3	
	P_*usefulness*5_	3.8	1.3	
Perceived ease of use	$P_{easeofuse1}$	3.6	1.3	0.959
(M = 3.6; SD =1.3)	$P_{easeofuse2}$	3.6	1.3	
Perceived Privacy	$P_{privacy1}$	3.5	1.3	0.868
(M = 3.6; SD =1.2)	$P_{privacy2}$	3.6	1.3	
Perceived Trust	$P_{trust1}$	3.6	1.3	0.962
(M = 3.5; SD = 1.2)	$P_{trust2}$	3.5	1.2	
	$P_{trust3}$	3.6	1.2	
Perceived Attitude	$P_{attitude1}$	3.7	1.3	0.99
(M = 3.7; SD = 1.3)	$P_{attitude2}$	3.7	1.3	
Perceived Intention	$P_{intention1}$	3.7	1.3	0.991
(M = 3.7; SD =1.3)	$P_{intention2}$	3.8	1.3	

**Table 4:** Cronbach’s Alpha Test Results If Item Deleted.

Attributes	Factors	${\mathrm{Average}}_{r}$	Raw-Alpha	Std.alpha
Perceived usefulness	$P_{usefulness1}$	0.74	0.92	0.92
	$P_{usefulness2}$	0.72	0.91	0.91
	$P_{usefulness3}$	0.70	0.90	0.90
	$P_{usefulness4}$	0.74	0.92	0.92
	$P_{usefulness5}$	0.73	0.92	0.92
Perceived Ease of use	$P_{easeofuse1}$	0.92	0.92	0.92
	$P_{easeofuse2}$	0.92	0.92	0.92
Perceived Privacy	$P_{privacy1}$	0.74	0.77	0.74
	$P_{privacy2}$	0.74	0.71	0.74
Perceived Trust	$P_{trust1}$	0.92	0.96	0.96
	$P_{trust2}$	0.89	0.94	0.94
	$P_{trust3}$	0.86	0.92	0.93
Perceived Attitude	$P_{attitude1}$	0.98	1.2	0.98
	$P_{attitude2}$	0.98	0.94	0.98
Perceived Intention	$P_{intention1}$	0.98	0.99	0.98
	$P_{intention2}$	0.98	0.97	0.98

${\mathrm{Average}}_{r}$: the average interitem correlation; raw-alpha: alpha based upon the covariances; std.alpha: the standardized alpha based upon the correlations.

**Table 5:** Construct reliability and validity.

	Cronbach’s Alpha	${\mathrm{Rho}}_{A}$	Composite Reliability (CA)	Average Variance Extracted (AVE)
Perceived usefulness	0.930	0.987	0.945	0.775
Perceived ease of use	0.958	0.959	0.979	0.959
Perceived Privacy	0.851	0.889	0.930	0.868
Perceived Trust	0.962	0.963	0.975	0.929
Perceived Attitude	0.990	0.990	0.995	0.990
Perceived Intention	0.991	0.991	0.995	0.991

**Table 6:** Discriminant validity (Fornell-Larcker Criterion).

	Perceived Attitude	PEU	Perceived Intention	Perceived Privacy	Perceived Trust	PU
Perceived Attitude	0.995					
PEU	0.545	0.979				
Perceived Intention	0.944	0.597	0.995			
Perceived Privacy	0.828	0.463	0.800	0.932		
Perceived Trust	0.908	0.568	0.927	0.887	0.964	
PU	0.509	0.879	0.532	0.453	0.521	0.881

## Data Availability

The original contributions presented in the study are included in the article, further inquiries can be directed to the corresponding author/s.
